# Association of *PON1*, *LEP* and *LEPR* Polymorphisms with Susceptibility to Breast Cancer: A Meta-Analysis

**DOI:** 10.31557/APJCP.2021.22.8.2323

**Published:** 2021-08

**Authors:** Soheila Sayad, Seyed Alireza Dastgheib, Meraj Farbod, Fatemeh Asadian, Mojgan Karimi-Zarchi, Seyedali Salari, Seyed Hossein Shaker, Jalal Sadeghizadeh-Yazdi, Hossein Neamatzadeh

**Affiliations:** 1 *Department of Surgery, Iran University of Medical Sciences, Tehran, Iran. *; 2 *Cancer Institute, Imam Khomeini Hospital, Tehran University of Medical Sciences, Tehran, Iran. *; 3 *Department of Medical Genetics, School of Medicine, Shiraz University of Medical Sciences, Shiraz, Iran. *; 4 *Department of Medical Laboratory Sciences, School of Paramedical Sciences, Shiraz University of Medical Sciences, Shiraz, Iran. *; 5 *Department of Obstetrics and Gynecology, Iran University of Medical Sciences, Tehran, Iran. *; 6 *Endometriosis Research Center, Iran University of Medical Sciences, Tehran, Iran. *; 7 *Department of Biology, Science and Arts University, Yazd, Iran. *; 8 *Department of Emergency Medicine, Iran University of Medical Sciences, Tehran, Iran. *; 9 *Department of Food Science and Technology, School of Public Health, Shahid Sadoughi University of Medical Sciences, Yazd, Iran. *; 10 *Department of Medical Genetics, Shahid Sadoughi University of Medical Sciences, Yazd, Iran. *; 11 *Mother and Newborn Health Research Center, Shahid Sadoughi University of Medical Sciences, Yazd, Iran. *

**Keywords:** Breast cancer, paraoxonase 1, leptin, leptin receptor, polymorphism

## Abstract

**Objective:**

Breast cancer is the most common cancer in American women, except for skin cancers. In this meta-analysis, the associations of polymorphisms within paraoxonase 1 (PON1), leptin (LEP) and leptin receptor (LEPR) genes with susceptibility to breast cancer were comprehensively evaluated.

**Methods:**

A universal search in PubMed, Scopus, CNKI, SID, Web of Knowledge and Google Scholar was performed to identify relevant studies up to 01 May, 2021. The strength of the associations was estimated by Odds ratios (ORs) with 95% confidence intervals (95% CIs).

**Results:**

A total of 39 case-control studies including 7 studies with 2005 cases and 2748 controls were on PON1 rs662, 6 studies with 2,031 cases and 1,973 controls on PON1 rs854560, 12 studies with 3,444 cases and 3,583 controls on LEP rs7799039, and 14 studies with 5,330 cases and 6,188 controls on LEPR rs1137101 were selected. Pooled data showed that PON1 rs662 and rs854560 polymorphisms were associated with risk of breast cancer in overall population, but not LEP rs7799039 and LEPR rs1137101.

**Conclusions:**

Our pooled data revealed that the PON1 rs662 and rs854560 polymorphisms were significantly associated with an increased risk of breast cancer in the overall population. However, LEP rs7799039 and LEPR rs1137101 polymorphisms were not associated.

## Introduction

Global facts and figures about the cancer revealed that breast cancer still key public health concern and leading cause of deaths among women globally (Jafari-Nedooshan et al., 2017; Moghimi et al., 2018). Heightened awareness of breast cancer risk in the past decades has led to an increase in the detection methods which can be used to detect the breast cancer in the early stages (Dinegde and Xuying, 2017). In the more affluent countries, mammography screening has been in place for a few decades and has successfully reduced mortality (Motamedi et al., 2012; Najminejad et al., 2020; Esmaeili et al., 2021). However, in developing countries, screening and paid little attention to fight with breast cancer is one of the lowest priorities in health policy makers (da Costa Vieira et al., 2017). Breast cancer is most likely triggered and/or promoted by multiple risk factors. The two strongest risk factors for breast cancer are gender and age (Feng et al., 2018). The etiological make-up of a heterogeneous and complex disease such as breast cancer is diverse and includes genetics and environmental factors. Breast cancer susceptibility gene 1 (BRCA1) and breast cancer susceptibility gene 2 (BRCA2) are the two major genes associated with hereditary breast and ovarian cancer (Forat-Yazdi et al., 2015; Neamatzadeh et al., 2015). However, there are more than 30 instances of SNPs identified as breast cancer susceptibility loci in the genome by GWAS (Kaklamani et al., 2011). Paraoxonase 1 (PON1), leptin (LEP) and leptin receptor (LEPR) genes are good example of a GWAS-identified locus that has been implicated in development of breast cancer (Gallicchio et al., 2007; Liu and Liu, 2011).

PON1, also called serum aromatic esterase 1, is the main means of protection of the nervous system against the neurotoxicity of organophosphates in serum (Richard et al., 2013; Mackness and Sozmen, 2020). Moreover, PON1 hydrolysis numerous exogenous and endogenous esters, such as arylesters, homocysteine thiolactone (HTL), other lactones, and cyclic carbonates (Costa et al, 2011; Seow et al., 2016). The human PON1 (MIM#602720) gene is a member of a multigene family consisting of three members including PON2 and PON3, which share ≈60% sequence identity with PON1 (Gallicchio et al., 2007; Liu and Liu, 2011). However, PON1 remains the most popular member of this family. The PON1 gene is located on chromosome 7q21.22, consisting 9 exons and spans 33.2 kb (Li et al., 1997). Of the PON1 polymorphisms, PON1 rs662 and rs854560 are most widely studied for their association with susceptibility to different cancers (Seow et al., 2016). Moreover, human LEP gene plays a critical role in energy expenditure as well as the progression of carcinogenesis (Tang et al., 2019). It is also reported that LEP may affect angiogenesis, inflammation, thrombosis, and tumor growth, invasion, and metastasis (Tang et al., 2019). It is revealed that the LEP signal may be transmitted through several signaling pathways such as JAK/STAT, MAPK, PI3K, Wnt/β-catenin, and ERK (Kavitha et al., 2013). The human LEP (MIM#164160) is located on chromosome 7q31.3, consists of three exons and spans approximately 16.4 kb (Funcke et al, 2014). It is highly polymorphic and the LEP rs7799039 G>A SNP is the most widely studied for its role in development of different human diseases (Tang et al., 2019). 

Over the past decade, several molecular epidemiological studies have been performed to identify the association of PON1 rs662, rs854560, LEP rs7799039G>A, and LEPR rs1137101 polymorphisms with susceptibility to breast cancer, but the findings have been conflicting. Thus, we performed a systematic review and updated meta-analysis to obtain a more precise assessment of the association between PON1, LEP and LEPR polymorphisms and the risk of breast cancer.

## Materials and Methods

*Search strategy*

This meta-analysis was reported based on the Preferred Reporting Items for Meta-analyses (PRISMA) guideline. In this meta-analysis, we carried out electronic literature retrieval in Medicine’s PubMed, Scopus, EMBASE, Web of Knowledge, Cochrane Library, Google Scholar, Scientific Information Database (SID), WanFang, VIP, Chinese Biomedical Database (CBD), Scientific Electronic Library Online (SciELO) and China National Knowledge Infrastructure (CNKI) database up to 01 May, 2021. The following keywords and terms were used to search: (‘’breast cancer’’ OR “breast tumor” OR “breast neoplasm” OR “breast malignant tumor” OR “breast carcinoma’’) AND (‘’ Paraoxonase 1’’ OR ‘’Serum Paraoxonase/Arylesterase’’ OR ‘’Serum Aryldialkylphosphatase’’ OR ‘’Aromatic Esterase’’ OR ‘’Arylesterase’’ OR ‘’A-Esterase’’ OR ‘’Esterase’’ OR ‘’PON1) AND (‘’Leptin’’ OR ‘’Obesity Factor’’ OR ‘’Obese Protein’’ OR ‘’LEP’’) AND (‘’Leptin Receptor’’ OR ‘’LEPR’’ OR ‘’OBR’’ ‘’OB Receptor’’ OR ‘’HuB219’’ OR ‘’CD295’’) AND (‘’Q192R’’ OR ‘’rs662’’ OR ‘’L55M’’ OR ‘’rs854560’’ OR ‘’LEP G2548A’’OR ‘’rs7799039’’ OR ‘’LEPR Q223R’’ OR ‘’rs1137101’’ OR ‘’LEPR Lys109Arg’’ OR ‘’rs1137100’’ OR ‘’rs1137101’’ OR ‘’c.668A>G’’ OR ‘’p.Gln223Arg’’ OR ‘’Arg223Gln’’ OR ‘’R223Q’’ OR ‘’Q223R’’ OR ‘’rs7799039’’ OR ‘’2548G/A’’) AND (‘’Gene’’ OR ‘’Genotype’’ OR ‘’Allele’’ OR ‘’Polymorphism’’ OR ‘’ Single nucleotide polymorphisms’’ OR ‘’SNP’’ OR ‘’Variation’’ OR ‘’Mutation’’). No restrictions were placed on the language, year of publication, ethnicity, and sample size. The references in included studies and reviewers were carefully checked for other potential data. When a publication involved some subgroups, it was treated separately.

*Selection and Exclusion Criteria*

The major selection criteria were as follows: 1) studies with case-control or cohort design; 2) studies that assessed the association of genetic variants within PON1, LEP and LEPR gene with risk of breast cancer; and (2) presented sufficient data to calculate the pooled-estimating. Accordingly, the major exclusion criteria were: 1) Studies did not evaluate the association of LEP, LEPR and PON1 polymorphisms and risk of breast cancer; 2) studies focusing on animals or in vitro; 3) Studies that did not provide usable or sufficient data for pooling; 4) case only studies or no controls; 5) linkage studies and family based studies (twins and sibling); 6) case reports, abstracts, comments, conference abstracts, editorials, reviews, meta-analysis; and 7) duplicated studies or data. When duplicated studies were published by the same author obtained from the same patient sample, only the one with the largest sample size was included in this meta-analysis.

*Data extraction*

Two authors independently extracted the data from each eligible study and if the extracted data was different, they would review the publication again and reached consensus. If they could not get a consistent assessment, third author would be invited to resolve the dispute and a final decision was made. The following data were extracted from each study: first author name, year of publication, country of origin, ethnicity (Asian, Caucasians, Africans and Mixed populations), numbers of cases and controls, source of control, genotype and allele frequencies, genotyping method, minor allele frequency (MAFs) and Hardy-Weinberg equilibrium (HWE) in controls.

*Statistical Analysis*

All of the statistical calculations were performed using Comprehensive Meta-Analysis (CMA) software version 2.0 (Biostat, USA). Two-sided P-values < 0.05 were considered statistically significant. The strength of association between genetic variants at PON1, LEP and LEPR genes and risk of breast cancer was estimated by Odds ratios (ORs) with 95% confidence intervals (95% CIs). The significance of the pooled effect size was determined by Z-test, in which P<0.05 was considered statistically significant. The associations was evaluated under all five genetic models, i.e., allele (B vs. A), homozygote (BB vs. AA), heterozygote (BA vs. AA), dominant (BB+BA vs. AA), and the recessive (BB vs. BA+AA), in which ‘’B’’ presents mutant and ‘’A’’ wild allele (Jafari-Nedooshan et al., 2019; Jafari et al., 2020). Between-study heterogeneity was estimated using a Cochran-based Q statistical test, with P-values less than 0.1 indicated the absence of indicated heterogeneity among studies. Moreover, a quantitative measure of between-study heterogeneity was tested using the I^2^ statistic (range of 0 to 100%), in which the heterogeneity was considered low, moderate, and high based on I^2^ values of 25%, 50%, and 75%, respectively. Thus, there was no heterogeneity (P > 0.1 or I^2^ < 50%) the fixed-effect model (Mantel-Haenszel method) was applied. There was heterogeneity (P <0.1 and I^2^ > 50%) the random-effect (DerSimonian-Laird method) model was used for analysis. Stratified analysis was carried out on the basis of ethnicity and source of controls. The Hardy-Weinberg equilibrium (HWE) for controls in each study was evaluated using the *χ*^2^ test and P >0.05 was considered to be consistent with HWE (Bahrami Dastgheib et al., 2020; Bahrami Shajari et al., 2020). To explore the influence of an individual study on the pooled data, sensitivity analysis was also used to confirm the stability of the results under all genetic models. Begg’s funnel plot test was used to assess possible publication bias, with P <0.05 being considered to present statistical significance.

## Results

*Selected Studies Characteristics*

**Figure 1:**
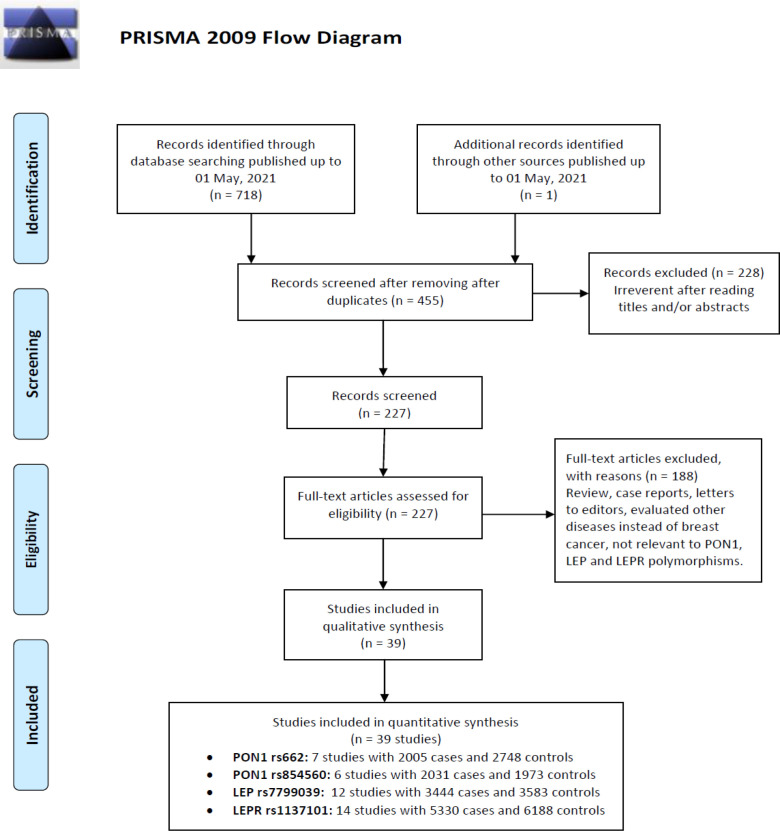
Flowchart of Literature Search and Selection Process

The selection process of eligible studies is presented in Figure 1. Initially, 719 studies were obtained through publication search in electronic databases and other sources. Irrelevant articles were excluded by evaluating the titles and abstracts. Therefore, 76 publications were deleted for obvious irrelevance. Finally, 39 case-control studies including 7 studies with 2005 cases and 2,748 controls were on PON1 rs662, 6 studies with 2,031 cases and 1,973 controls on PON1 rs854560, 12 studies with 3,444 cases and 3,583 controls on LEP rs7799039, and 14 studies with 5,330 cases and 6,188 controls on LEPR rs1137101 were selected. Pooled data showed that PON1 rs662 and rs854560 polymorphisms were associated with risk of breast cancer in overall population, but not LEP rs7799039 and LEPR rs1137101. Table 1 describes principal characteristics of included studies. The studies have been carried out in USA, Brazil, Italy, Malaysia, Egypt, turkey, China, Iran, Mexico, Sri Lanka, India, Tunisia, Nigeria, and Korea. Among these studies, eight studies were conducted among Asians, two studies among Caucasians and two studies Africans. Seven different genotyping methods were used: PCR, PCR-RFLP, TaqMan, SNPstream, and TOFMS. The genotype, allele and minor allele frequency (MAF) in each study for PON1 rs662, rs854560, LEP rs7799039 and LEPR rs1137101 are shown in Table 1. Moreover, the distribution of genotypes in the controls was in agreement with Hardy-Weinberg equilibrium (HWE) for all selected studies, except for one study on IL-8 -251T>A polymorphism (Table 1).

*Quantitative Data Synthesis*

*PON1 rs662*

Table 2 listed the main results of the meta-analysis of PON1 rs662 polymorphism and breast cancer risk. When all the eligible studies were pooled into the meta-analysis, a significant association was found between PON1 rs662 and breast cancer under all three genetic models, i.e., allele (G vs. A: OR= 0.719, 95% CI: 0.648-0.798; p≤0.001, Figure 1A), homozygote (GG vs. AA: OR= 0.542, 95% CI: 0.332-0.885; p=0.014) and dominant (GG+GA vs. AA: OR= 0.720, 95% CI: 0.330-0.864; p=0.011). When subgroup analysis by ethnicity performed the results showed that the PON1 rs662 polymorphism was associated with breast cancer risk among Caucasian women under two genetic models, i.e., homozygote (GG vs. AA: OR= 0.341, 95% CI: 0.134-0.866; p=0.024) and dominant (GG+GA vs. AA: OR= 0.317, 95% CI: 0.119-0.839; p=0.021), but not among Asians. Moreover, subgroup analysis by source of controls showed that the variant was associated with breast cancer in PB group of studies.

*PON1 rs854560*

**Figure 2:**
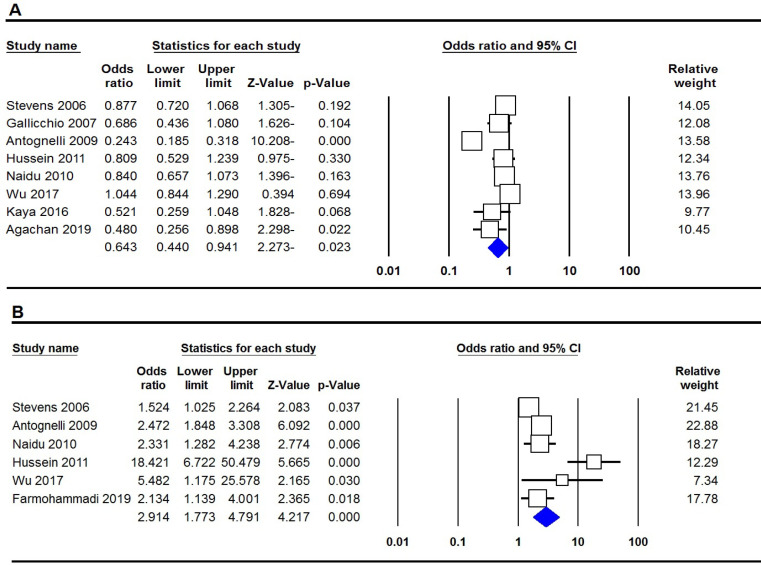
Forest Plot for Association of the PON1 Polymorphisms with Breast Cancer Risk in Overall Population. A, rs662 (allele model); B, rs854560 (homozygote model)

Table 2 listed the main results of the meta-analysis of PON1 rs854560 polymorphism and breast cancer risk. Pooled data showed that the PON1 rs854560 polymorphism was significantly associated with risk of breast cancer under all four genetic models, i.e., allele (A vs. T: OR=2.107, 95% CI: 1.401-3.167; p≤0.001), homozygote (AA vs. TT: OR= 3.214, 95% CI: 1.757-5.879; p≤0.001, Figure 2B), heterozygote (AT vs. TT: OR= 0.379, 95% CI: 0.208-0.691; p=0.002), dominant (AA+AT vs. TT: OR= 1.868, 95% CI: 1.293-2.700; p=0.001) and recessive (AA vs. AT+TT: OR= 3.067, 95% CI: 1.687-5.575; p≤0.001). Subgroup analysis by ethnicity revealed that PON1 rs854560 polymorphism was a significantly associated with breast cancer among Asian and Caucasian women.

*LEPR rs1137101*

Table 2 listed the main results of the meta-analysis of LEPR rs1137101 polymorphism and breast cancer risk. When all the eligible studies were pooled into the meta-analysis, no significant association was found between LEPR rs1137101 and breast cancer under all five genetic models in overall population. Subgroup analysis by ethnicity revealed that the variant was a significantly associated with breast cancer among African women under all four genetic models, i.e., allele (A vs. G: OR= 0.772, 95% CI: 1.161-1.654; p≤0.001), homozygote (AA vs. GG: OR= 0.772, 95% CI: 1.339-2.786; p≤0.001), heterozygote (AG vs. GG: OR= 0.772, 95% CI: 1.010-1.772; p=0.043), and dominant (AA+AG vs. GG: OR= 0.772, 95% CI: 1.268-2.137; p≤0.001), but not among Caucasians and Asians.

*LEP rs7799039G>A*

Table 2 listed the main results of the meta-analysis of LEP rs7799039G>A polymorphism and breast cancer risk. Pooled data showed that this polymorphism was not associated with risk of breast cancer under all four genetic models in overall population. Moreover, subgroup analysis by ethnicity and source of controls revealed that LEP rs7799039G>A polymorphism was not significantly associated with breast cancer.

*Test of Heterogeneity and sensitivity analyses*

As shown in Tables 2 and 4, there was a significant heterogeneity existed under most genetic models for PON1 rs662, rs854560, LEP rs7799039 and LEPR rs1137101 polymorphisms. Thus, stratified analyses by ethnicity and source of controls carried out to find the potential source of heterogeneity. Results showed that ethnicity and source of controls have overall effect on the heterogeneity for these polymorphisms. We carried out the sensitivity analyses to assess the robustness of the results by removing each study in turn and all the results were not essentially altered, suggesting that the results of the present meta-analysis were statistically stable.

*Publication bias*

**Figure 3:**
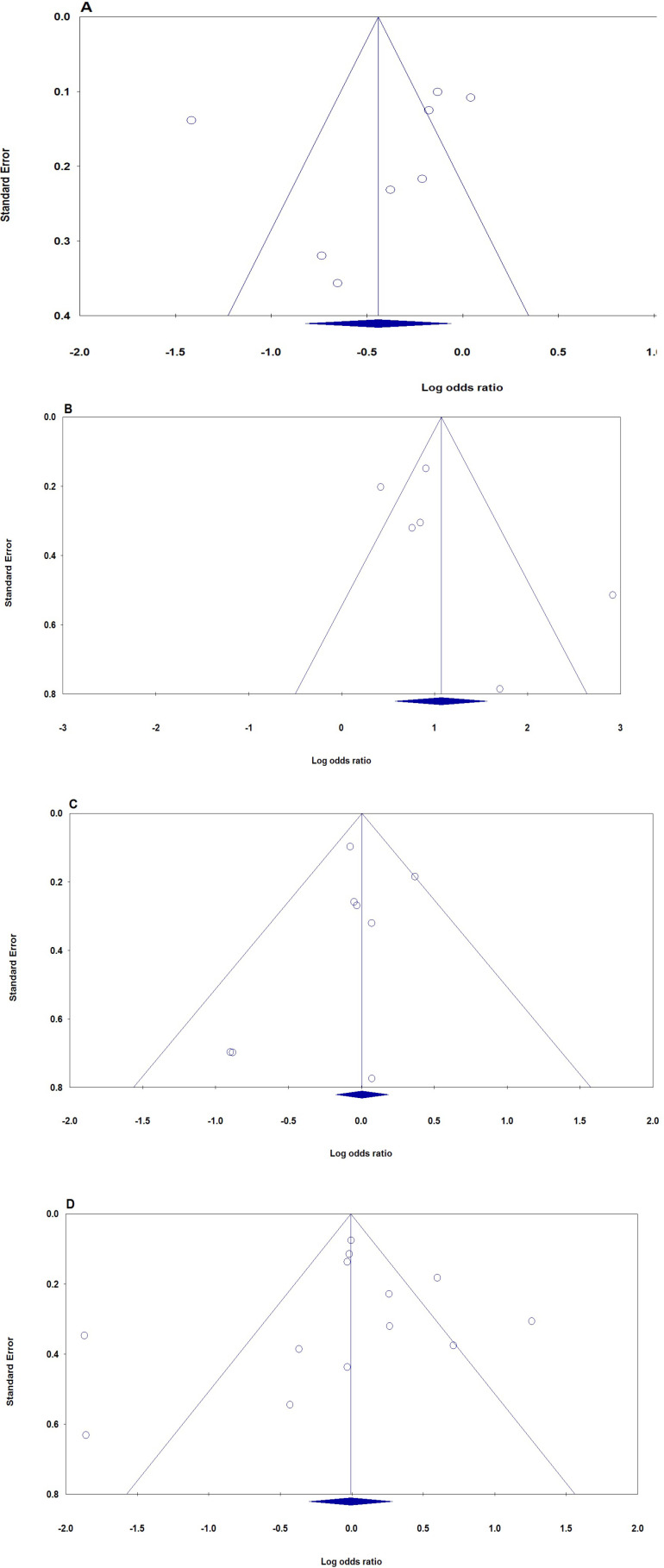
The Funnel Plots of Publication Bias for Association of the PON1, LEP and LEPR Polymorphism with Breast Cancer Risk in Overall Population. A: PON1 rs662 (allele mode); B: rs854560 (homozygote model), C: LEP rs7799039 (heterozygote model), and D: LEPR rs1137101 (dominant model)

The publication bias of the studies was evaluated using the funnel plot and Egger’s test. Publication bias was not seen in the funnel plot (Figure 3). No statistically significant difference was discovered in the Egger’s test for PON1 rs662, rs854560, LEP rs7799039 and LEPR rs1137101 polymorphisms, indicating low publication bias in the current meta-analysis. Moreover, funnel plots’ shape of all comparison models did not reveal any obvious evidence of asymmetry and all P values of Egger’s tests were more than 0.05, providing statistical evidence of funnel plots’ symmetry.

**Table 1:** Characteristics of the Case-Control Studies Included in the Meta-Analyses

First Author	Country (Ethnicity)	SOC	Genotyping Method	Cases/ controls	Cases					Controls				
					Genotype			Allele		Genotype			Allele	
PON1 rs662					AA	AG	GG	A	G	AA	AG	GG	A	G
Stevens 2006	USA (Caucasian)	PB	PCR-RFLP	483/483	259	182	42	700	266	238	198	47	674	292
Gallicchio 2007	Brazil (Mixed)	PB	PCR-RFLP	58/904	38	15	5	91	25	469	353	82	1291	517
Antognelli 2009	Italy (Caucasian)	PB	PCR-RFLP	547/544	484	50	13	1018	76	340	152	52	832	256
Naidu 2010	Malaya (Asian)	HB	PCR-RFLP	387/252	200	158	29	558	216	115	115	22	345	159
Hussein 2011	Egypt (African)	PB	PCR-RFLP	100/100	51	41	8	143	57	46	42	12	134	66
Kaya 2016	Turkey (Caucasian)	HB	TaqMan	32/35	10	11	11	31	33	5	13	17	23	47
Wu 2017	China (Asian)	HB	TaqMan	365/378	155	156	54	466	264	167	156	55	490	266
Agachan 2019	Turkey (Caucasian)	PB	PCR-RFLP	33/52	17	4	12	38	28	6	29	17	41	63
PON1 rs854560					TT	TA	AA	T	A	TT	TA	AA	T	A
Stevens 2006	USA (Caucasian)	PB	PCR-RFLP	483/493	176	230	77	582	384	202	233	58	637	349
Antognelli 2009	Italy (Caucasian)	PB	PCR-RFLP	547/607	107	115	325	329	765	188	188	231	564	650
Naidu 2010	Malaya (Asian)	HB	PCR-RFLP	387/269	159	178	50	496	278	126	126	17	378	160
Hussein 2011	Egypt (African)	PB	PCR-RFLP	100/76	19	21	60	59	141	35	35	6	105	47
Wu 2017	China (Asian)	HB	TaqMan	483/483	284	72	9	640	90	346	30	2	722	34
Farmohammadi 2019	Iran (Asian)	HB	PCR-RFLP	150/150	47	65	38	159	141	66	59	25	191	109
LEP rs7799039					GG	GA	AA	G	A	GG	GA	AA	G	A
Snoussi 2006	Tunisia (Caucasian)	HB	PCR-RFLP	308/222	37	152	119	226	390	11	99	112	37	152
Vairaktaris 2008	Greece (Caucasian)	HB	PCR	150/152	32	78	40	142	158	112	99	11	32	78
Teras 2009	USA (Caucasian)	PB	SNPstream	1077/1086	445	445	187	1335	819	442	442	202	445	445
Cleveland 2010	USA (Caucasian)	PB	PCR	1059/1101	226	492	341	944	1174	180	561	360	226	492
Morris 2013	Mexico (Mixed)	HB	PCR	130/189	22	71	37	115	145	46	95	48	22	71
Rostami 2015	Iran (Asian)	HB	PCR-RFLP	203/171	115	64	24	294	112	63	77	31	115	64
Mahmoudi 2015	Iran (Asian)	PB	PCR-RFLP	45/41	27	11	7	65	25	17	19	5	27	11
Karakus 2015	Turkey (Caucasian)	PB	PCR	199/185	49	105	45	203	195	47	98	40	49	105
Mohammadzadeh 2015	Iran (Asian)	HB	PCR-RFLP	100/100	36	55	9	127	73	52	45	3	36	55
Rodrigo 2017	Sri Lanka (Asian)	PB	PCR	80/80	32	43	5	107	53	53	24	3	32	43
Liu 2018	China (Asian)	HB	TOFMS	434/442	-	182	252	-	686	-	206	236	-	182
Geriki 2019	India (Asian)	HB	PCR-RFLP	93/186	15	45	33	75	111	54	75	57	15	45
LEPR rs1137101					AA	AG	GG	A	G	AA	AG	GG	A	G
Snoussi 2006	Tunisia (African)	NS	PCR-RFLP	308/222	98	145	65	341	275	102	90	30	294	150
Woo 2006	Korea (Asian)	HB	PCR	45/45	0	12	33	12	78	0	8	37	8	82
Gallicchio 2007	USA (Caucasian)	PB	TaqMan	53/872	14	24	15	52	54	278	443	151	999	745
Han 2008	China (Asian)	HB	PCR	240/500	33	41	166	107	373	12	78	410	102	898
Okobia 2008	Nigeria (African)	HB	PCR-RFLP	209/209	46	107	56	199	219	56	107	46	219	199
Teras 2009	USA (Caucasian)	PB	SNP stream	648/659	128	332	181	588	694	125	314	211	564	736
Cleveland 2010	USA (Caucasian)	PB	PCR	1059/1098	173	521	355	867	1231	187	551	360	925	1271
Nyante 2011	USA (Caucasian)	PB	PCR	1972/1775	494	952	526	1940	2004	416	847	485	1679	1817
Kim 2012	Korea (Asian)	HB	PCR	390/447	8	88	294	104	676	6	91	350	103	791
Mohammadzadeh 2014	Iran (Asian)	HB	PCR-RFLP	100/100	25	56	19	106	94	54	40	6	148	52
Mahmoudi 2015	Iran (Asian)	PB	PCR-RFLP	45/41	19	25	1	63	27	17	18	6	52	30
Wang 2015	China (Asian)	PB	PCR-RFLP	150/128	20	25	105	65	235	3	19	106	25	231
Rodrigo 2017	Sri Lanka (Asian)	PB	PCR-RFLP	80/80	65	9	6	139	21	60	6	14	126	34
El-Hussiny 2017	Egypt (African)	NS	PCR-RFLP	48/79	24	15	9	63	33	22	24	2	68	28

SOC, Source Of Controls; HB, Hospital Based; PB, Population Based; RFLP, Restriction Fragment Length Polymorphism; MAF, Minor Allele Frequency; HWE, Hardy-Weinberg Equilibrium

**Table 2:** Meta-Analysis Results of Association between PON1 rs662 Polymorphism and Breast Cancer Risk

Polymorphism	Genetic Model	Type of Model	Heterogeneity		Odds Ratio				Publication Bias	
			I^2^ (%)	P_H_	OR	95% CI	Z_test_	P_OR_	P_Beggs_	P_Eggers_
Overall	G vs. A	Random	91.39	≤0.001	0.719	0.648-0.798	-6.234	≤0.001	0.063	0.467
	GG vs. AA	Random	73.8	≤0.001	0.542	0.332-0.885	-2.446	0.014	0.035	0.221
	GA vs. AA	Fixed	14.09	0.32	1.011	0.800-1.278	0.092	0.926	0.901	0.374
	GG+GA vs. AA	Random	90.55	≤0.001	0.534	0.330-0.864	-2.554	0.011	0.107	0.428
	GG vs. GA+AA	Random	62.05	0.01	0.72	0.492-1.053	-1.696	0.09	0.173	0.576
Ethnicity										
Caucasian	G vs. A	Random	94.69	≤0.001	0.48	0.220-1.047	-1.846	0.065	1	0.694
	GG vs. AA	Random	82.08	0.001	0.341	0.134-0.866	-2.262	0.024	1	0.479
	GA vs. AA	Random	57.75	0.069	0.894	0.481-1.661	-0.355	0.723	0.734	0.486
	GG+GA vs. AA	Random	93.93	≤0.001	0.317	0.119-0.839	-2.312	0.021	1	0.578
	GG vs. GA+AA	Random	78.94	0.003	0.594	0.278-1.269	-1.345	0.179	0.734	0.872
Asian	G vs. A	Fixed	41.99	0.189	0.951	0.810-1.116	-0.617	0.537	NA	NA
	GG vs. AA	Fixed	0	0.378	0.943	0.663-1.341	-0.326	0.744	NA	NA
	GA vs. AA	Fixed	0	0.952	1.027	0.721-1.462	0.146	0.884	NA	NA
	GG+GA vs. AA	Fixed	50.42	0.159	0.931	0.751-1.153	-0.658	0.511	NA	NA
	GG vs. GA+AA	Fixed	0	0.607	0.959	0.688-1.337	-0.247	0.805	NA	NA
Source of Controls										
HB	G vs. A	Fixed	54.8	0.109	0.923	0.789-1.079	-1.01	0.313	0.296	0.332
	GG vs. AA	Fixed	36.58	0.207	0.878	0.625-1.233	-0.75	0.453	0.296	0.105
	GA vs. AA	Fixed	0	0.918	1.05	0.750-1.470	0.284	0.777	0.296	0.163
	GG+GA vs. AA	Fixed	52.79	0.12	0.904	0.732-1.117	-0.933	0.351	0.296	0.429
	GG vs. GA+AA	Fixed	0	0.514	0.907	0.662-1.243	-0.609	0.543	0.296	0.007
PB	G vs. A	Random	93.28	≤0.001	0.563	0.311-1.020	-1.895	0.058	0.462	0.793
	GG vs. AA	Random	77.27	0.001	0.445	0.215-0.920	-2.186	0.029	0.462	0.645
	GA vs. AA	Fixed	49.26	0.096	0.976	0.704-1.353	-0.147	0.883	0.22	0.354
	GG+GA vs. AA	Random	92.42	≤0.001	0.429	0.209-0.878	-2.316	0.021	0.462	0.657
	GG vs. GA+AA	Random	73.1	0.005	658	0.351-1.232	-1.307	0.191	0.806	0.96

**Table 3:** Meta-Analysis Results of Association between PON1 rs854560 Polymorphism and Breast Cancer Risk

Polymorphism	Genetic Model	Type of Model	Heterogeneity		Odds Ratio				Publication Bias	
			I^2^ (%)	P_H_	OR	95% CI	Z_test_	P_OR_	P_Beggs_	P_Eggers_
Overall	A vs. T	Random	92.43	≤0.001	2.107	1.401-3.167	3.582	≤0.001	0.22	0.21
	AA vs. TT	Random	81.73	≤0.001	3.214	1.757-5.879	3.789	≤0.001	0.462	0.27
	AT vs. TT	Random	81.85	≤0.001	0.379	0.208-0.691	-3.17	0.002	1	0.478
	AA+AT vs. TT	Random	81.83	≤0.001	1.868	1.293-2.700	3.326	0.001	0.22	0.12
	AA vs. AT+TT	Random	84.64	≤0.001	3.067	1.687-5.575	3.674	≤0.001	0.462	0.375
Ethnicity										
Asian	A vs. T	Random	82.54	0.003	1.785	1.150-2.772	2.581	0.01	0.296	0.248
	AA vs. TT	Fixed	0	0.536	2.387	1.573-3.622	4.09	≤0.001	1	0.152
	AG vs. TT	Random	77.65	0.011	0.792	0.313-2.001	-0.493	0.622	1	0.751
	AA+AT vs. TT	Random	93.29	≤0.001	1.212	0.469-3.132	0.397	0.691	1	0.802
	AA vs. AT+TT	Fixed	0	0.442	2.043	1.383-3.016	3.592	≤0.001	0.296	0.317
Caucasian	A vs. T	Random	93.83	≤0.001	1.56	0.941-2.587	1.725	0.085	NA	NA
	AA vs. TT	Fixed	73.12	0.054	2.086	1.650-2.638	6.143	≤0.001	NA	NA
	AG vs. TT	Random	79.06	0.029	0.559	0.331-0.946	-2.169	0.03	NA	NA
	AA+AT vs. TT	Random	79.34	0.028	1.491	0.987-2.253	1.897	0.058	NA	NA
	AA vs. AT+TT	Fixed	81.38	0.02	1.878	1.134-3.109	2.45	0.014	NA	NA
Source of Controls										
HB	A vs. T	Random	82.54	0.003	1.785	1.150-2.772	2.581	0.01	0.296	0.248
	AA vs. TT	Random	90.35	≤0.001	3.48	1.455-8.321	2.804	0.005	1	0.478
	AG vs. TT	Random	90.91	≤0.001	0.316	0.131-0.761	-2.567	0.01	1	0.501
	AA+AT vs. TT	Random	93.29	≤0.001	1.212	0.469-3.132	0.397	0.691	1	0.802
	AA vs. AT+TT	Random	92.04	≤0.001	3.359	1.432-7.879	2.785	0.005	1	0.566
PB	A vs. T	Random	95.14	≤0.001	2.254	1.228-4.140	2.622	0.009	1	0.482
	AA vs. TT	Random	90.35	≤0.001	3.48	1.455-8.321	2.804	0.005	1	0.478
	AG vs. TT	Random	90.91	≤0.001	0.316	0.131-0.761	-2.567	0.01	1	0.501
	AA+AT vs. TT	Random	82.19	0.004	1.84	1.137-2.976	2.483	0.013	0.296	0.413
	AA vs. AT+TT	Fixed	0	0.465	2.038	1.380-3.009	3.58	≤0.001	0.296	0.33

**Table 4:** Meta-Analysis Results of Association between LEP rs7799039 Polymorphism and Breast Cancer Risk

Polymorphism	Genetic Model	Type of Model	Heterogeneity		Odds Ratio				Publication Bias	
			I^2^ (%)	P_H_	OR	95% CI	Z_test_	P_OR_	P_Beggs_	P_Eggers_
Overall	A vs. G	Random	82.4	≤0.001	0.98	0.761-1.263	-0.154	0.878	0.265	0.51
	AA vs. GG	Random	71.3	0.001	0.905	0.572-1.432	-0.428	0.669	0.386	0.405
	AG vs. GG	Fixed	12.18	0.335	0.993	0.857-1.150	-0.095	0.924	0.901	0.57
	AA+AG vs. GG	Random	84.71	≤0.001	0.931	0.600-1.446	-0.319	0.75	0.386	0.48
	AA vs. AG+GG	Random	46.09	0.072	0.931	0.723-1.197	-0.56	0.576	0.71	0.506
Ethnicity										
Asian	A vs. G	Random	89.62	≤0.001	1.086	0.538-2.191	0.23	0.818	0.734	0.458
	AA vs. GG	Random	76.03	0.006	1.303	0.404-4.203	0.443	0.658	0.308	0.092
	AG vs. GG	Fixed	0	0.424	0.832	0.506-1.368	-0.724	0.469	1	0.294
	AA+AG vs. GG	Random	90.89	≤0.001	1.051	0.393-2.810	0.099	0.921	0.734	0.609
	AA vs. AG+GG	Fixed	52.94	0.095	0.914	0.576-1.450	-0.382	0.702	0.308	0.068
Caucasian	A vs. G	Random	69.07	0.039	0.847	0.675-1.063	-1.431	0.153	1	0.815
	AA vs. GG	Random	71.32	0.031	0.672	0.390-1.157	-1.434	0.151	1	0.759
	AG vs. GG	Fixed	56.67	0.099	1.014	0.863-1.190	0.164	0.869	1	0.646
	AA+AG vs. GG	Fixed	64.42	0.06	0.708	0.466-1.076	-1.616	0.106	1	0.837
	AA vs. AG+GG	Fixed	64.81	0.058	0.903	0.776-1.052	-1.31	0.19	1	0.747
Source of Controls										
HB	A vs. G	Random	90.52	≤0.001	1.037	0.542-1.986	0.11	0.913	0.296	0.497
	AA vs. GG	Random	85.4	0.001	1.286	0.377-4.386	0.402	0.688	0.296	0.501
	AG vs. GG	Fixed	0	0.443	0.94	0.638-1.385	-0.313	0.754	1	0.259
	AA+AG vs. GG	Random	90.74	≤0.001	1.09	0.412-2.886	0.173	0.863	0.846	0.066
	AA vs. AG+GG	Random	67.46	0.046	1.089	0.530-2.240	0.232	0.816	1	0.582
PB	A vs. G	Random	74.44	0.008	1.067	0.771-1.477	0.391	0.696	0.734	0.477
	AA vs. GG	Fixed	21.62	0.281	0.82	0.658-1.023	-1.761	0.078	0.308	0.175
	AG vs. GG	Fixed	0	0.711	0.919	0.770-1.096	-0.945	0.345	0.734	0.53
	AA+AG vs. GG	Random	84.25	≤0.001	1.019	0.552-1.880	0.059	0.953	0.734	0.525
	AA vs. AG+GG	Fixed	0	0.842	1	0.847-1.180	-0.005	0.996	0.089	0.025

**Table 5:** Meta-Analysis Results of Association between LEPR rs1137101 Polymorphism and Breast Cancer Risk

Polymorphism	Genetic Model	Type of Model	Heterogeneity		Odds Ratio				Publication Bias	
			I^2^ (%)	P_H_	OR	95% CI	Z_test_	P_OR_	P_Beggs_	P_Eggers_
Overall	A vs. G	Random	86.48	≤0.001	0.943	0.780-1.139	-0.614	0.539	0.661	0.781
	AA vs. GG	Random	84.75	≤0.001	0.928	0.631-1.365	-0.379	0.705	0.76	0.867
	AG vs. GG	Random	74.12	≤0.001	0.991	0.763-1.289	-0.064	0.949	0.669	0.717
	AA+AG vs. GG	Random	83.33	≤0.001	0.994	0.742-1.331	-0.041	0.967	0.427	0.761
	AA vs. AG+GG	Random	76.74	≤0.001	0.965	0.767-1.214	-0.302	0.763	0.745	0.867
Ethnicity										
Caucasian	A vs. G	Fixed	41.76	0.161	0.977	0.916-1.043	-0.688	0.491	0.734	0.349
	AA vs. GG	Fixed	41.86	0.16	0.96	0.842-1.094	-0.615	0.539	0.734	0.285
	AG vs. GG	Fixed	0	0.92	0.983	0.873-1.107	-0.287	0.774	1	0.202
	AA+AG vs. GG	Fixed	0	0.859	0.996	0.892-1.112	-0.071	0.944	1	0.273
	AA vs. AG+GG	Fixed	55.12	0.083	0.976	0.883-1.079	-0.468	0.639	0.734	0.431
Asian	A vs. G	Random	89.54	≤0.001	0.711	0.419-1.207	-1.264	0.206	1	0.907
	AA vs. GG	Random	87.83	≤0.001	0.442	0.124-1.570	-1.262	0.207	1	0.762
	AG vs. GG	Random	86.29	≤0.001	0.749	0.273-2.054	-0.562	0.574	0.452	0.394
	AA+AG vs. GG	Random	90.55	≤0.001	0.595	0.201-1.758	-0.939	0.347	0.452	0.397
	AA vs. AG+GG	Random	72.07	0.001	0.664	0.413-1.066	-1.697	0.09	0.763	0.888
African	A vs. G	Fixed	3.035	0.357	1.386	1.161-1.654	3.612	≤0.001	1	0.813
	AA vs. GG	Fixed	2.894	0.358	1.931	1.339-2.786	3.52	≤0.001	1	0.598
	AG vs. GG	Fixed	61.34	0.075	1.337	1.010-1.772	2.026	0.043	0.296	0.11
	AA+AG vs. GG	Fixed	0	0.423	1.647	1.268-2.137	3.747	≤0.001	1	0.916
	AA vs. AG+GG	Fixed	63.26	0.066	1.845	0.997-3.415	1.949	0.051	0.296	0.199
Source of Controls										
HB	A vs. G	Random	93.07	≤0.001	0.931	0.504-1.721	-0.227	0.82	0.806	0.83
	AA vs. GG	Random	93.33	≤0.001	0.964	0.209-4.445	-0.047	0.963	1	0.815
	AG vs. GG	Random	90.34	≤0.001	0.866	0.285-2.629	-0.254	0.799	0.734	0.597
	AA+AG vs. GG	Random	93.72	≤0.001	0.836	0.230-3.041	-0.272	0.786	0.734	0.651
	AA vs. AG+GG	Random	81.48	≤0.001	0.963	0.567-1.635	-0.141	0.888	0.806	0.43
PB	A vs. G	Random	86.15	≤0.001	0.816	0.666-0.999	-1.967	0.049	0.465	0.244
	AA vs. GG	Random	72.44	0.001	0.893	0.758-1.051	-1.361	0.173	0.367	0.271
	AG vs. GG	Fixed	8.403	0.364	0.979	0.871-1.100	-0.359	0.719	0.763	0.753
	AA+AG vs. GG	Fixed	40.64	0.12	0.974	0.875-1.086	-0.471	0.638	0.133	0.23
	AA vs. AG+GG	Random	67.54	0.005	0.882	0.699-1.112	-1.063	0.288	0.367	0.249

## Discussion

Genetics play an important role in development and progression breast cancer (Yazdi et al., 2015). There are more and more association studies searching susceptibility genes involved in breast cancer. To date, several variants within PON1 gene associated with susceptibility to breast cancer have been verified. rs662 and rs854560 polymorphism are the most characterized SNPs that are associated with development this disease. Our present work indicated that both rs662 and rs854560 polymorphisms at PON1 gene were associated with an increased risk of BC in the overall population. All previous meta-analysis have indicated that PON1 rs662 was associated with risk of breast cancer, but not rs854560. Two meta-analysis by Fang et al., (2012) and Saadat (2012) suggested that the PON1 rs662 is a risk factor for the development of breast cancer. Wu et al., (2017) evaluated the associations of PON1 rs662 and rs854560 polymorphisms with risk of breast cancer in 365 cases and 378 controls from the Guangxi region of southern China. Their results showed that PON1 rs854560 genetic polymorphisms may be associated with the risk of BC. However, they have found that rs662 polymorphism was not associated with breast cancer risk, or with any of the clinicopathological parameters. Pan et al., (2019) in meta-analysis reported that the PON1 rs662 is associated with decrease of breast cancer risk. Their results showed an increased risk in the Caucasian and Asian population as well as HB group and PB group. However, there was an association between rs854560 polymorphism and increased breast cancer risk. Liu et al., (2019) in a mate-analysis revealed that PON1 rs854560 polymorphism could be used to identify individual with elevated susceptibility to breast cancer. However, they have not found any positive association between PON1 rs662 polymorphism and breast cancer in polled analyses. In other meta-analysis, Zhang et al., (2015) found that PON1 rs662 polymorphism was associated with a decreased risk in breast cancer. Our meta-analysis supports the growing body of evidence that the PON1 rs662 and rs854560 polymorphisms is emerging as a RISK factor for breast cancer.

Our pooled data indicated that LEP rs7799039 variant was not associated with risk of breast cancer in overall population and ethnicity. Liu and Liu (2011) in a meta-analysis based on three studies with 2,003 cases and 1,967 controls revealed for LEP rs7799039G>A polymorphism and nine studies with 4,627 cases and 5,476 controls for LEPR rs1137101 revealed that these polymorphisms were not associated with breast cancer risk. However, Yan et al., (2016) in a meta-analysis suggests that the LEP rs7799039G>A plays an important role in breast cancer susceptibility, especially in Caucasian. Although previous meta-analyses have reported the association between rs7799039 and LEPR rs1137101 polymorphisms and susceptibility to breast cancer, the current meta-analysis was more in the number of studies included and larger in sample size, which comparatively reduced the influence of contingency on the pooled data. Therefore, our conclusions were more persuasive and accurate than previous meta-analysis.

The current meta-analysis has several limitations. Therefore, some conclusions of this study should be cautiously interpreted. First, only a small number of studies were found on PON1 polymorphisms. Further studies are still required to confirm the relationship of these polymorphisms with breast cancer in different populations, especially in African and mixed populations. Second, in this work there was a considerable heterogeneity in overall population studies. Differences of ethnicity, genotyping methods and source of controls may partially explain the significant heterogeneity. Moreover, various adjusted confounders, different study designs, and other undetected factors may also lead to the presence of heterogeneity. Finally, none of the included studies separately analyzed the relations of different confounders such as age, lifestyle, family history, hormone therapy, etc. in addition, breast cancer is a complex disease which is influenced by the environment, genetic factors, and genotype-environment interactions. Thus, these interactions in development of breast cancer should be considered.

In summary, this meta-analysis aimed to summarize association between the PON1 rs662, rs854560 LEP rs7799039 and LEPR rs1137101 polymorphisms and susceptibility to breast cancer. The pooled data revealed that rs662 and rs854560 polymorphisms were associated with risk of BC and could potentially serve as useful genetic markers for breast cancer. However, there was no association between LEP rs7799039 and LEPR rs1137101 polymorphisms and breast cancer risk. More studies among different ethnicities are required to be done to reinforce the results of the current study. Nevertheless, gene-gene or gene-environment interaction which is closely related to development of breast cancer should be considered in future studies.

## Author Contribution Statement

Soheila Sayad, Meraj Farbod: conceptualization, investigation. Seyed Alireza Dastgheib, Mojgan Karimi-Zarchi: Software, original draft preparation. Seyedali Salari, Seyed Hossein Shaker: Investigation. Fatemeh Asadian: Investigation, writing. Fatemeh Asadian, Hossein Neamatzadeh: Methodology, software. Seyed Alireza Dastgheib: Formal analysis, investigation. Seyed Hossein Shaker: Project administration. Jalal Sadeghizadeh-Yazdi, Hossein Neamatzadeh: Writing, reviewing, editing

## Data Availability

The datasets generated during and/or analyzed during this study are available from the corresponding author on reasonable request.

## References

[B1] Bahrami R, Dastgheib SA, Niktabar SM (2020). Association of BMP4 rs17563 polymorphism with nonsyndromic cleft lip with or without cleft palate risk: Literature Review and Comprehensive Meta-Analysis. Fetal Pediatr Pathol.

[B2] Bahrami R, Shajari A, Aflatoonian M (2020). Association of REarranged during Transfection (RET) c 73 + 9277T > C and c 135G > a polymorphisms with susceptibility to hirschsprung disease: A Systematic Review and Meta-Analysis. Fetal Pediatr Pathol.

[B3] Costa LG, Giordano G, Furlong CE (2011). Pharmacological and dietary modulators of paraoxonase 1 (PON1) activity and expression: The hunt goes on. Biochemical Pharmacol.

[B4] da Costa Vieira RA, Biller G, Uemura G, Ruiz CA, Curado MP (2017). Breast cancer screening in developing countries. Clinics.

[B5] Dinegde NG, Xuying L (2017). Awareness of breast cancer among female care givers in tertiary cancer hospital, China. Asian Pac J Cancer Prev.

[B6] Esmaeili R, Mohammadi S, Jafarbeik-Iravani N (2021). Expression of SCUBE2 and BCL2 predicts favorable response in ERα positive breast cancer. Arch Iran Med.

[B7] Fang DH, Fan CH, Ji Q (2012). Differential effects of paraoxonase 1 (PON1) polymorphisms on cancer risk: Evidence from 25 published studies. Mol Biol Rep.

[B8] Feng Y, Spezia M, Huang S (2018). Breast cancer development and progression: Risk factors, cancer stem cells, signaling pathways, genomics, and molecular pathogenesis. Genes Dis.

[B9] Forat-Yazdi M, Neamatzadeh H, Sheikhha MH, Zare-Shehneh M, Fattahi M (2015). BRCA1 and BRCA2 common mutations in Iranian breast cancer patients: A meta analysis. Asian Pac J Cancer Prev.

[B10] Funcke J-B, von Schnurbein J, Lennerz B (2014). Monogenic forms of childhood obesity due to mutations in the leptin gene. Mol Cell Pediat.

[B11] Gallicchio L, McSorley MA, Newschaffer CJ (2007). Body mass, polymorphisms in obesity-related genes, and the risk of developing breast cancer among women with benign breast disease. Cancer Detect Prev.

[B12] Jafari-Nedooshan J, Dastgheib SA, Kargar S (2019). Association of IL-6 -174 G>C Polymorphism with Susceptibility to Colorectal Cancer and Gastric Cancer: a Systematic Review and Meta-Analysis. Acta Med (Hradec Kralove).

[B13] Jafari-Nedooshan J, Kargar S, Neamatzadeh H (2017). Lack of association of the fat mass and obesity associated (FTO) gene rs9939609 polymorphism with breast cancer Risk: a Systematic Review and Meta-Analysis Based on Case - Control Studies. Asian Pac J Cancer Prev.

[B14] Jafari M, Jarahzadeh MH, Dastgheib SA (2020). Association of PAI-1 rs1799889 polymorphism with susceptibility to ischemic stroke: a Huge Meta-Analysis based on 44 Studies. Acta Med (Hradec Kralove).

[B15] Kaklamani V, Yi N, Sadim M (2011). The role of the fat mass and obesity associated gene (FTO) in breast cancer risk. BMC Med Genet.

[B16] Kavitha K, Kowshik J, Kishore TKK, Baba AB, Nagini S (2013). Astaxanthin inhibits NF-κB and Wnt/β-catenin signaling pathways via inactivation of Erk/MAPK and PI3K/Akt to induce intrinsic apoptosis in a hamster model of oral cancer. Biochimica et Biophysica Acta - General Subjects.

[B17] Li WF, Matthews C, Disteche CM, Costa LG, Furlong CE (1997). Paraoxonase (Pon1) gene in mice: Sequencing, chromosomal localization and developmental expression. Pharmacogenetics.

[B18] Liu C, Liu L (2011). Polymorphisms in three obesity-related genes (LEP, LEPR, and PON1) and breast cancer risk: a meta-analysis. Int Soc Oncodevelopment Biol Med.

[B19] Liu P, Wang Q, Cui Y, Wang J (2019). A meta-analysis of the relationship between paraoxonase 1 polymorphisms and cancer. Free Radical Res.

[B20] Mackness M, Sozmen EY (2020). A critical review on human serum paraoxonase-1 in the literature: Truths and misconceptions. Turk J Biochem.

[B21] Moghimi M, Kargar S, Jafari MA (2018). Angiotensin converting enzyme insertion/deletion polymorphism is associated with breast cancer risk: A Meta-Analysis. Asian Pac J Cancer Prev.

[B22] Motamedi S, Majidzadeh K, Mazaheri M (2012). Tamoxifen resistance and CYP2D6 copy numbers in breast cancer patients. Asian Pac J Cancer Prev.

[B23] Najminejad H, Farhadihosseinabadi B, Dabaghian M (2020). Key regulatory miRNAs and their interplay with mechanosensing and mechanotransduction signaling pathways in breast cancer progression. Mol Cancer Res.

[B24] Neamatzadeh H, Shiryazdi SM, Kalantar SM (2015). BRCA1 and BRCA2 mutations in Iranian breast cancer patients: A systematic review. J Res Med Sci.

[B25] Pan X, Huang L, Li M (2019). The association between PON1 (Q192R and L55M) gene polymorphisms and risk of cancer: A Meta-Analysis Based on 43 Studies. Bio Med Res Int.

[B26] Richard SA, Frank EA, D’Souza CJM (2013). Correlation between cholinesterase and paraoxonase 1 activities: Case series of pesticide poisoning subjects. BioImpacts.

[B27] Saadat M (2012). Paraoxonase 1 genetic polymorphisms and susceptibility to breast cancer: A meta-analysis. Cancer Epidemiol.

[B28] Seow DCC, Gao Q, Yap P (2016). Profile of the paraoxonase 1 (PON1) gene 192Q/R polymorphism and clinical associations among older Singaporean Chinese with Alzheimer’s and Mixed Dementia. Dementia Geriatric Cognitive Disorders Extra.

[B29] Tang W, Kang M, Liu C, Qiu H (2019). Leptin rs7799039 (G2548A) polymorphism is associated with cancer risk: a meta-analysis involving 25,799 subjects. OncoTargets Ther.

[B30] Yan W, Ma X, Gao X, Zhang S (2016). Association between leptin (-2548G/A) genes polymorphism and breast cancer susceptibility: A meta-Analysis. Medicine (United States).

[B31] Yazdi MF, Rafieian S, Gholi-Nataj M (2015). CYP2D6 Genotype and risk of recurrence in tamoxifen treated breast cancer patients. Asian Pac J Cancer Prev.

[B32] Zhang M, Xiong H, Fang L (2015). Paraoxonase 1 (PON1) Q192R gene polymorphism and cancer risk: A meta-analysis based on 30 publications. Asian Pac J Cancer Prev.

